# Validation of a Handheld 6-Lead Device for QT Interval Monitoring in Resource-Limited Settings

**DOI:** 10.1001/jamanetworkopen.2024.15576

**Published:** 2024-06-07

**Authors:** John Z. Metcalfe, Tamsin Economou, Fahd Naufal, Murat Kucukosmanoglu, Robert Kleiman, Patrick P. J. Phillips, Francesca Conradie

**Affiliations:** 1 Division of Pulmonary and Critical Care Medicine, Zuckerberg San Francisco General Hospital and Trauma Center, University of California, San Francisco; 2 Department of Clinical Medicine, University of the Witwatersrand, Johannesburg, South Africa; 3 D-Prime LLC, McLean, Virginia; 4 Clario Inc, Philadelphia, Pennsylvania

## Abstract

**Importance:**

Rifampin-resistant tuberculosis treatment regimens require electrocardiographic (ECG) monitoring due to the use of multiple QTc-prolonging agents. Formal 12-lead ECG devices represent a significant burden in resource-constrained clinics worldwide and a potential barrier to treatment scale-up in some settings.

**Objective:**

To evaluate the diagnostic accuracy of a handheld 6-lead ECG device within resource-constrained clinics.

**Design, Setting, and Participants:**

This diagnostic study was performed within a multicenter, pragmatic (broad eligibility criteria with no exclusions for randomized participants), phase 3 rifampin-resistant tuberculosis treatment trial (BEAT Tuberculosis [Building Evidence for Advancing New Treatment for Tuberculosis]) in South Africa. A total of 192 consecutive trial participants were assessed, and 191 were recruited for this substudy between January 21, 2021, and March 27, 2023. A low proportion (3 of 432 [0.7%]) of all screened trial participants were excluded due to a QTc interval greater than 450 milliseconds. Triplicate reference standard 12-lead ECG results were human calibrated with readers blinded to 6-lead ECG results.

**Main Outcomes and Measures:**

Diagnostic accuracy, repeatability, and feasibility of a 6-lead ECG device.

**Results:**

A total of 191 participants (median age, 36 years [IQR, 28-45 years]; 81 female participants [42.4%]; 91 participants [47.6%] living with HIV) with a median of 4 clinic visits (IQR, 3-4 visits) contributed 2070 and 2015 12-lead and 6-lead ECG assessments, respectively. Across 170 participants attending 489 total clinic visits where valid triplicate QTc measurements were available for both devices, the mean 12-lead QTc measurement was 418 milliseconds (range, 321-519 milliseconds), and the mean 6-lead QTc measurement was 422 milliseconds (range, 288-574 milliseconds; proportion of variation explained, *R*^2^ = 0.4; *P* < .001). At a QTc interval threshold of 500 milliseconds, the 6-lead ECG device had a negative predictive value of 99.8% (95% CI, 98.8%-99.9%) and a positive predictive value of 16.7% (95% CI, 0.4%-64.1%). The normal expected range of within-individual variability of the 6-lead ECG device was high (±50.2 milliseconds [coefficient of variation, 6.0%]) relative to the 12-lead ECG device (±22.0 milliseconds [coefficient of variation, 2.7%]). The mean (SD) increase in the 12-lead QTc measurement during treatment was 10.1 (25.8) milliseconds, with 0.8% of clinic visits (4 of 489) having a QTc interval of 500 milliseconds or more.

**Conclusions and Relevance:**

This study suggests that simplified, handheld 6-lead ECG devices are effective triage tests that could reduce the need to perform 12-lead ECG monitoring in resource-constrained settings.

## Key Points

**Question:**

What is the diagnostic accuracy and feasibility of a handheld 6-lead electrocardiographic (ECG) device for QTc interval measurement in a resource-limited setting?

**Findings:**

In this diagnostic study, 2070 longitudinal reference standard 12-lead ECG measurements and 2105 6-lead measurements were evaluated in a nested prospective cohort of 191 participants in a tuberculosis trial. At a QTc interval threshold of 500 milliseconds, the handheld 6-lead device had a high negative predictive value of 99.8% but a low positive predictive value of 16.7%.

**Meaning:**

This study suggests that a 6-lead ECG device is an effective triage test and could reduce the need to perform 12-lead ECG monitoring in resource-limited settings.

## Teaser

This diagnostic study evaluates the diagnostic accuracy, repeatability, and feasibility of a handheld 6-lead electrocardiographic device for QTc interval measurement, relative to formal standard 12-lead electrocardiographic devices, within resource-constrained settings.

## Introduction

In 2022, the World Health Organization estimated that 410 000 of 10.6 million cases of tuberculosis (TB) globally were multidrug- or rifampin-resistant (RR) TB.^1^ Relative to patients with drug-susceptible TB, patients with RR-TB have worse treatment outcomes, different clinical and social characteristics, and, historically, a higher risk of TB medication adverse events. Although shorter-course regimens containing new or repurposed medicines, such as bedaquiline, pretomanid, delamanid, clofazimine, and fluoroquinolones, have an overall adverse event rate approaching that of drug-susceptible TB treatment,^2,3,4^ they confer a rare but difficult-to-predict risk for torsade de pointes, a distinctive form of polymorphic ventricular tachycardia. Electrocardiographic (ECG) monitoring of the QT interval is therefore recommended at baseline and monthly (or more frequent) treatment intervals.^5^

The ECG QT interval represents the time necessary for ventricular depolarization and repolarization, with prolongation of the QT interval beyond 500 milliseconds associated with increasing risk of torsade de pointes.^6,7,8^ Modern electrocardiography, the physiological basis and recording format for which was established in 1902 with a string galvanometer,^9^ remains the criterion standard risk assessment for predicting drug-related serious cardiac events. However, performing 12-lead ECG measurements requires a consistent power supply, specialized equipment and consumables, and personnel to accurately place leads,^10^ limiting clinic capacity and presenting a barrier to scale up newer RR-TB regimens in some settings.^11^

Recently, highly portable handheld ECG devices able to accurately represent the 6 limb leads of formal ECG machines and assess cardiac rate and rhythm, atrioventricular conduction, and interval duration measurements (including QTc) have been developed.^12^ Although lacking the chest (ie, precordial) leads crucial for diagnosing myocardial ischemia, chamber size, and arrhythmogenic disease (eg, Brugada syndrome), such devices may improve data storage and transfer, reduce personnel time and costs, and facilitate serial ECG measurements when needed.

We performed serial handheld 6-lead ECG measurements within the phase III BEAT Tuberculosis trial (Building Evidence for Advancing New Treatment for Tuberculosis [NCT04062201]). Our principal objectives were to assess the diagnostic accuracy, repeatability, and feasibility of handheld 6-lead ECG QTc interval measurements relative to formal standard 12-lead ECG measurements to inform their clinical and research utility in a resource-limited, routine RR-TB treatment setting.

## Methods

We undertook a diagnostic prospective cohort study nested within the BEAT Tuberculosis trial.^13^ BEAT Tuberculosis is a multicenter open-label, phase 3 trial to establish the efficacy and safety of a 6-month regimen consisting of bedaquiline, delamanid, linezolid, levofloxacin, and clofazimine, with retention of either clofazimine or levofloxacin depending on results of culture-based fluoroquinolone susceptibility tests, compared with a 9-month bedaquiline- and clofazimine-containing standard of care regimen for RR-TB. BEAT Tuberculosis in South Africa was designed as a pragmatic trial with broad eligibility criteria: participants were 12 years of age or older and had received a diagnosis of RR-TB by testing with GeneXpert Ultra (Cepheid). Relevant exclusion criteria were treatment with more than 28 days of prior second-line TB drugs, clinically significant ECG measurement abnormality, or baseline QTcF interval (QT interval corrected for heart rate by the Fridericia cube root formula) greater than 450 milliseconds (this was changed to >480 milliseconds on October 20, 2020, to liberalize trial recruitment)^13^ according to the in-clinic automated (ie, “computer read”) 12-lead ECG algorithm. Participants agreeing to participate in this substudy provided a separate informed consent and were included between January 21, 2021, and March 27, 2023. Ethical approval was obtained from the University of Witwatersrand Human Research Ethics Committee, the South African Health Products Regulatory Authority, and the University of California, San Francisco Human Research Protection Program. This study followed the Standards for Reporting of Diagnostic Accuracy (STARD) reporting guideline.

### Reference Standard 12-Lead ECG Measurements

Twelve-lead ECG measurements (ELI 150; Welch Allyn) were recorded at baseline and weeks 4, 12, and 24 (corresponding to pharmacokinetic sampling time points in BEAT Tuberculosis) in triplicate at approximate 5-minute intervals within a 15-minute period with participants at rest in the supine position. Electrocardiographic measurements were done at approximately the same time of day throughout the study as far as possible (to account for diurnal variation in QT interval) and at approximately 4 to 6 hours after dose, before lunch (approximate time needed for study TB drugs to reach the maximum concentration). The 12-lead ECG measurements were recorded by trained clinic nurses and transferred digitally to a secure, centralized ECG core laboratory at Clario Inc for analysis.

At Clario, QTcF intervals were measured using computer-assisted caliper placements on 3 consecutive beats. Trained analysts reviewed all ECG devices for correct lead and beat selection and adjusted the placement of algorithm-placed calipers as necessary using a proprietary-validated electronic caliper system. The tangent method was used to determine the T-wave offset. Lead consistency was used to maintain uniformity in measurements between participants with difficult T-wave morphologic characteristics.^14^ A cardiologist then verified the QTc interval and performed an analysis of morphologic characteristics. Electrocardiogram readers were blinded to participant identifiers, treatment, and details of the study. Recordings were 10 seconds, with a sampling rate of 1000 samples per second. Interval duration measurements for the 12-lead ECGs were performed on the unfiltered Lead II whenever possible; the secondary measurement lead was V5, and the tertiary measurement lead was V2. Mean values were generated from the individual ECG measurements; at the beat level, the QT interval was corrected based on the preceding RR interval, generating a beat-level QTc interval. The mean of the 3 beat-level QTc values was then reported as the mean QTc value for each individual 12-lead ECG measurement.

### Six-Lead ECG Measurements

We used the KardiaMobile 6L (AliveCor),^15^ a small (9.0 cm × 3.0 cm × 0.72 cm), commercial handheld ECG device consisting of a single sensor with 3 stainless steel electrodes allowing for 3 points of contact and generation of a 6-lead ECG measurement (recorded leads I and II; derived lead III [from the Einthoven triangle]; and derived leads aVF, aVR, and aVL [from the Goldberger central terminal]). Triplicate 6-lead ECG recordings were performed with the participant in the sitting position (eFigure 1 in Supplement 1) within 15 minutes after 12-lead ECG recordings. Recordings were 30 to 120 seconds in duration, had a sampling rate of 300 samples per second with approximately 30 seconds between individual measurements, and were transmitted via Bluetooth to a study smartphone (iPhone 6, iOS 12.1; Apple Inc). The KardiaMobile device calculates the QT interval using a US Food and Drug Administration (FDA)–approved, clinically validated EK12 QT algorithm^16,17^ that assesses 10 seconds of waveform data at 5-second intervals. A median QT interval is derived from these 5 separate measurements. The device algorithm assesses QTc from lead II after filtering; when lead II was not analyzable, the secondary measurement lead was lead I, and the tertiary measurement lead was lead III.

### Statistical Analysis

Heart rate, QT interval, and QTc interval were abstracted from reference standard 12-lead (ie, manually read Clario measurements) and automated or algorithmic handheld 6-lead ECG measurements. The QTc interval was calculated using the Fridericia formula (QTc = QT/RR^1/3^)^18^; we further assessed a population-specific (QTcN)^19^ and the Bazett (QTcB = QT/RR^1/2^) correction method. The mean increase in QTc interval was measured using a linear mixed-effects regression model of the QTc interval on the clinic visit with random intercepts by participant.^20,21^ Clinic visits with fewer than triplicate 12-lead reference standard QTcF measurements were excluded from the primary analysis. Visit-level triplicate (or more, if >3 measurements were available) QTcF measurements for both devices were averaged, compared, and illustrated using scatterplots.^20^ Outlier 12-lead reference standard QTcF measurements (a range of >40 milleseconds between triplicate measurements) were rechecked and reanalyzed if appropriate by an adjudicating cardiologist (R.K.). As in prior studies,^21,22^ we reported the maximum mean change in QTc interval from baseline.

Quantitative intermodality agreement was measured using Bland-Altman plots (eMethods in Supplement 1).^23^ Contingency tables were used to describe the performance of the QTcF interval classification model using the FDA-suggested cut points of 500 and 480 milleseconds.^24,25,26^ We assessed repeatability using a linear mixed-effects model fit to the numerical QTcF values. Such models allow for the global estimation of the within-individual SD optimally weighted for the correlation structure of the repeated measurements.^27,28^ Assuming that 95% of measurements are located within 1.96 SDs, the normal expected range of within-individual test repeatability can be calculated by expressing this SD as a percentage of the individual’s mean QTcF interval. Thus, given an underlying “true” mean test result, linear mixed-effect models estimate the expected variability that will occur with repeated measurements. Because test-retest differences were not normally distributed, the 12-lead and 6-lead datasets were verified as having 95% of differences contained within 1.96 SDs of their mean values by a resampling procedure using 10 000 bootstrap iterations. We conducted a qualitative survey of nurses who performed the 12-lead and handheld 6-lead measurements, assessing ease of use and preferences for use in clinical workflow. All *P* values were 2-sided, and results were deemed statistically significant at *P* < .05. Data analysis was performed using Python, version 3.11.1 (Python Software Foundation) and Stata, version 15.1 (StataCorp LLC).

## Results

### Study Population

**Figure 1.**
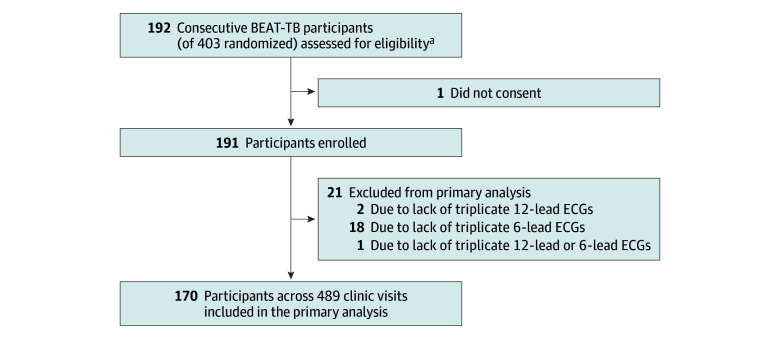
Study Enrollment The primary analysis included only participants with 3 or more measurements from both 12-lead reference standard and handheld 6-lead electrocardiographic (ECG) devices at each clinic visit. BEAT-TB indicates Building Evidence for Advancing New Treatment for Tuberculosis. ^a^Of 432 participants assessed for eligibility in the parent trial, only 3 (0.7%) were excluded for a QTcF (Fridericia-corrected QT) interval greater than 450 milliseconds.

Of the 432 total participants assessed for eligibility in the BEAT Tuberculosis trial, none were excluded for clinically significant ECG measurement abnormality, and only 3 (0.7%) were excluded for a QTcF interval greater than 450 milliseconds. Of 403 total participants randomized in BEAT Tuberculosis, 192 consecutive participants were assessed, and 191 were recruited for this substudy (Figure 1). The median age was 36 years (IQR, 28-45 years), 81 participant (42.4%) were female, 110 participants (57.6%) were male, and 91 participants (47.6%) were living with HIV (Table 1).^29,30,31^ One-hundred eighty-eight participants (98.4%) across 697 total clinic visits (median, 4 visits per participant [IQR, 3-4 visits per participant]) had reference standard triplicate 12-lead ECG measurements across a median of 6 months (IQR, 6-6 months) of treatment and formed our analysis population. A total of 94 participants (49.2%) were randomized to the intervention arm and assigned a median of 4 (IQR, 3-4) concurrent QT-prolonging drugs (bedaquiline, delamanid, levofloxacin, and clofazimine); participants in the control arm were administered a median of 3 (IQR, 3-3) QT-prolonging drugs (bedaquiline, levofloxacin, and clofazimine). Participants were receiving nonstudy RR-TB treatment regimens a median of 8 days (IQR, 4-14 days) prior to randomization.

**Table 1.** Characteristics of the Study Population

Characteristic	Patients included in substudy (N = 191)
Age, median (range), y	36 (13-69)
Sex, No. (%)	
Female	81 (42.4)
Male	110 (57.6)
HIV, No.	91 (47.6)
Antiretroviral regimens, No. (%)	
Efavirenz^a^	50 (54.9)
Lopinavir or ritonavir^b^	13 (14.3)
Randomized to intervention arm, No. (%)	94 (49.2)
Baseline weight	
Median (range), kg	53.3 (30.1-91.0)
BMI, median (range)	18.8 (12.5-34.6)
Baseline potassium, median (range), mEq/L	4.5 (3.2-6.3)
Baseline QTcF interval, mean of triplicates	
Median (range), ms	411 (328-486)
Concurrent QT-prolonging anti-TB medications in BEAT-TB, No. (%)^c^	
4	59 (30.9)
3	131 (68.6)
2	1 (0.5)
Total weeks on treatment, median (range)	
Clofazimine	24.4 (0.0-79.0)
Bedaquiline	25.7 (0.0-80.9)
Delamanid	24.1 (0.0-80.3)
Levofloxacin	25.6 (0.0-80.9)
QTcF interval ≥500 ms during treatment, No. (%)^d^	4 (2.1)

Abbreviations: BEAT-TB, Building Evidence for Advancing New Treatment for Tuberculosis; BMI, body mass index (calculated as weight in kilograms divided by height in meters squared); QTcF, QT interval corrected for heart rate by the Fridericia cube root formula; TB, tuberculosis.

SI conversion factors: To convert potassium to millimoles per liter, multiply by 1.0.

^a^ Efavirenz can cause QT interval prolongation but currently lacks evidence for a risk of torsade de pointes when taken as recommended.^29,30^ The denominator is the number of participants with HIV (n = 91).

^b^ Lopinavir or ritonavir is primarily a concern due to a drug-drug interaction with bedaquiline, delamanid, and clofazimine; it can cause QT interval prolongation but currently lacks evidence for a risk of torsade de pointes when taken as recommended.^31^ Nine participants had antiretroviral regimens coded as including both lopinavir or ritonavir and efavirenz. The denominator is the number of participants with HIV (n = 91).

^c^ Drugs noted were taken at any point in treatment and not necessarily throughout; the highest number of medications taken concurrently is noted for each participant. Treatment regimens included combinations of the QT-prolonging medication bedaquiline, delamanid, levofloxacin, and clofazimine. Of 131 participants taking regimens with 3 QT interval–prolonging medications, 86 (65.6%) were taking bedaquiline, levofloxacin, and clofazimine; 26 (19.8%) were taking bedaquiline, delamanid, and levofloxacin; and 15 (11.5%) were taking bedaquiline, delamanid, and clofazimine.

^d^ One additional participant experienced sudden death 3 months after treatment completion and had QTcF measurements exceeding 500 milliseconds during preceding treatment visits; the exact cause of death is unknown.

### Diagnostic Accuracy of the 6-Lead ECG Device

**Figure 2.**
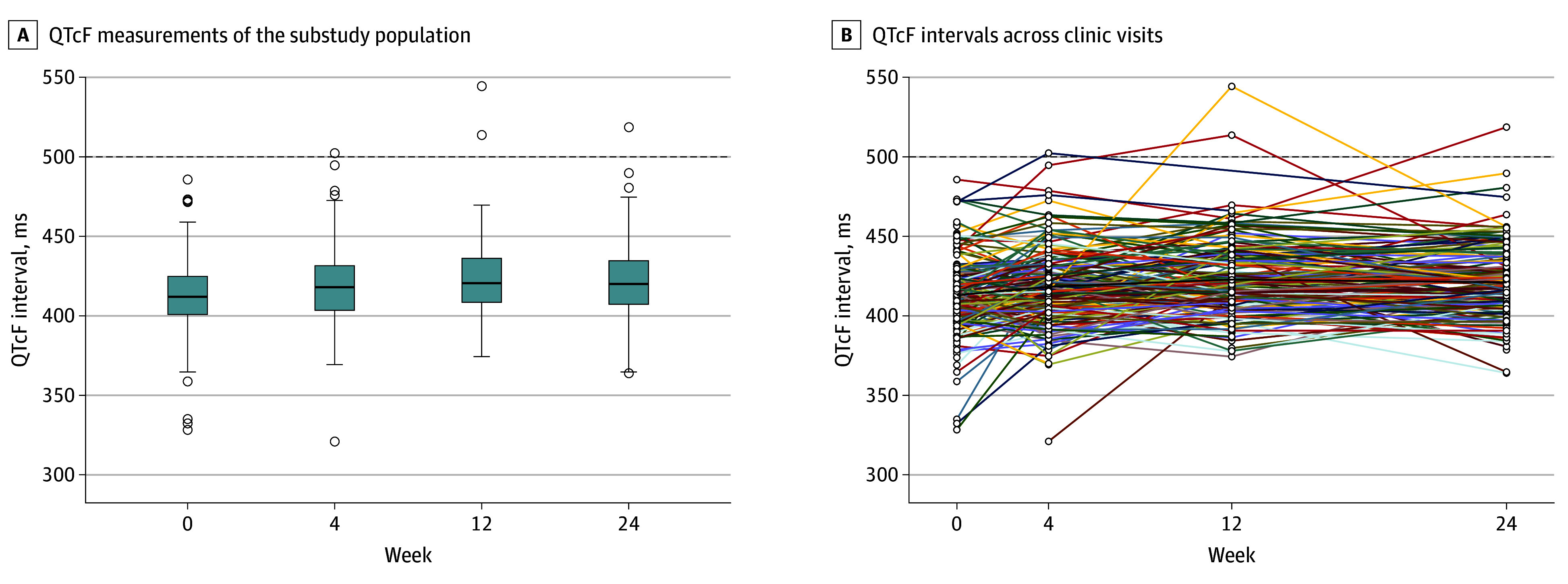
Change in Fridericia-Corrected QT (QTcF) Interval Across Clinic Visits A, Medians, quartiles, and 95% CIs (vertical bars) for QTcF interval measurements of the substudy population at baseline through week 24 (end of study treatment). Dots indicate outliers. B, Participant-level QTcF interval line plot across clinic visits, with the mean QTcF interval indicated as a black line. The dashed line indicates the 500-millisecond threshold. Both median and mean QTcF intervals increased between the baseline and week 12 study visit.

Over the study duration, 2070 12-lead ECG assessments and 2015 6-lead ECG assessments were made. A total of 43 of the 2070 12-lead assessments (2.1%) and 235 of the 2015 6-lead assessments (11.7%) were indeterminate (eTable 1 in Supplement 1). One participant (0.5%) had a 12-lead triplicate mean QTcF interval of less than 480 milliseconds at baseline, and 4 participants (2.1%) developed a QTcF interval of 500 milliseconds or more during treatment. Across 170 participants attending 489 total clinic visits where valid triplicate QTcF measurements were available for both devices (eTable 2 in Supplement 1), the mean 12-lead QTcF interval was 418 milliseconds (range, 321-519 milliseconds), and the mean 6-lead QTcF interval was 422 milliseconds (range, 288-574 milliseconds [proportion of variation explained, *R*^2^ = 0.4; *P* < .001]) (Figure 2). Reference standard 12-lead QTcF measurements were a mean of 4.5 milliseconds (95% CI, 2.9-6.0 milliseconds) shorter than autocalculated 12-lead QTcF measurements (ie, those printed on the ECG header and used for trial purposes). The mean (SD) QTcF interval as measured by the 12-lead ECG device increased by 10.1 (25.8) milliseconds (*P* < .001), and the mean (SD) QTcF interval as measured by the 6-lead ECG device increased by 9.9 (44.6) milliseconds (*P* = .002) during treatment (Figure 2). In addition, in this trial with concurrent administration of up to 4 QT-prolonging anti-TB drugs, 97.9% of patients’ QTc measurements (187 of 191) remained below 500 milliseconds over a 6-month treatment period.

**Figure 3.**
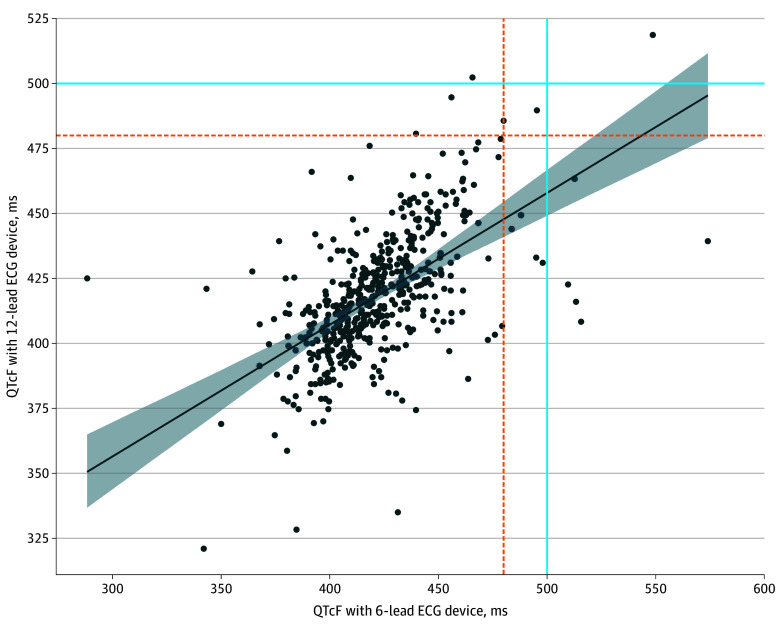
Association Between Handheld 6-Lead Electrocardiographic (ECG) Device and Reference Standard 12-Lead ECG Device Fridericia-Corrected QT (QTcF) Measurements A scatterplot shows the association between handheld 6-lead ECG device and reference standard 12-lead ECG device QTcF measurements for all triplicate averaged 6-lead and 12-lead measurements across all clinic visits as independent events (n = 489), along with 95% CI of the regression line. The shaded area indicates the 95% CI. The regression coefficient between the *z* score–standardized 6-lead and 12-lead measurements was 0.6 (proportion of variation explained, *R*^2^ = 0.4; *P* < .001). Dashed lines indicate thresholds of 480 milliseconds, and solid lines indicate thresholds of 500 milliseconds.

At a diagnostic cut point of 500 milliseconds, 98.8% of clinic visits (483 of 489) were correctly classified by the handheld 6-lead device: 482 as true negatives (ie, QTc interval <500 milliseconds measured by both devices) and 1 as true positive (ie, QTc interval of ≥500 milliseconds measured by both devices). Of 4 clinic visits (0.8% of total) with a 12-lead QTcF interval of 500 milliseconds or more, 2 were excluded from the primary analysis due to lack of triplicate 6-lead measurements (1 with duplicate 6-lead QTc interval of a mean (SD) of 377 (11) milliseconds and 1 with a single 6-lead measurement of 395 milliseconds); of the 2 clinic visits included in the primary analysis, 1 had false-negative results (triplicate 6-lead: 465.7 milliseconds) (Figure 3). The 6-lead device further classified 5 QTcF intervals of 500 milliseconds or more where the 12-lead QTcF interval was less than 500 milliseconds (ie, false positives), with a mean (SD) difference of 95.1 (31.1) milliseconds. Therefore, a 6-lead QTc measurement of less than 500 milliseconds was associated with the absence of a 12-lead QTcF measurement of 500 milliseconds or more (negative predictive value [NPV], 99.8% [95% CI, 98.8%-99.9%]); a 6-lead QTc measurement of 500 milliseconds or greater was less predictive of the presence of a 12-lead QTcF measurement of 500 milliseconds or more (positive predictive value [PPV], 16.7% [95% CI, 0.4%-64.1%]). Sensitivity was 50.0% (95% CI, 1.3%-98.7%), and specificity was 99.0% (95% CI, 97.6%-99.7%). At a 480-millisecond QTcF threshold, the 6-lead device had 4 false negatives (mean [SD] difference, −30.5 [16.6] milliseconds) and 9 false positives (mean [SD] difference, 75.9 [33.0] milliseconds) for an NPV of 99.2% (95% CI, 97.9%-99.8%) and a PPV of 18.2% (95% CI, 2.3%-51.8%). Diagnostic accuracy was worse when fewer than triplicate 6-lead ECG measurements were available at a clinic visit (eTable 3 in Supplement 1). Results stratified by sex, age (<50 years vs ≥50 years), and body mass index (calculated as weight in kilograms divided by height in meters squared; underweight [≤18.5] vs normal [>18.5 and ≤25]) are shown in eTable 4 in Supplement 1.

### Inherent Variability of the 6-Lead ECG Device

The normal expected range of variability (ie, how much it would be expected to vary on repeated measurements under identical conditions) was ±22.0 milliseconds (coefficient of variation, 2.7%) for the reference standard 12-lead ECG device and ±50.2 milliseconds (coefficient of variation, 6.0%) for the handheld 6-lead ECG device (Table 2). Within-visit concordance of ECG measurement interpretations was greater for the 12-lead ECG device (98.8% [643 of 651 visits]) compared with the 6-lead ECG device (92.9% [487 of 524 visits]) (eTable 5 in Supplement 1).

**Table 2.** Handheld 6-Lead and Reference Standard 12-Lead ECG Device QTcF Interval Comparative Statistics and Device-Specific Parameters^a^

Characteristic	12-lead ECG device QTcF interval, ms	6-lead ECG device QTcF interval, ms	Difference (12-lead measurement–6-lead measurement), ms
Comparative statistics			
No. of clinic visits^b^	489	489	NA
Mean (SD)	418 (23.8)	422 (27.3)	−3.6 (24.0)
95% CI	416 to 420	420 to 424	−5.8 to −1.6
Median (range)	417 (321 to 519)	420 (288 to 574)	−3.0 (−135.0 to 137.0)
Individual device parameters			
No. of clinic visits^b^	651	524	NA
Normal expected range^c^	±22.0	±50.2	NA
Repeatability, SD^d^	±11.2	±25.6	NA
ICC (SE)^e^	0.8 (0.01)	0.5 (0.03)	NA

Abbreviations: ECG, electrocardiographic; ICC, intraclass correlation coefficient; NA, not applicable; QTcF, QT interval corrected for heart rate by the Fridericia cube root formula.

^a^ Summary statistics compare the triplicate averaged 12-lead and 6-lead ECG measurements from clinic visits where these measurements were available for each device. Individual device parameters were calculated for each device from visits including triplicate averaged measurements, regardless of measurement availability for the other device.

^b^ Number of clinic visits with triplicate averaged 12-lead or 6-lead ECG measurements.

^c^ Normal expected range of within-individual variability in QTcF interval, estimated using linear mixed-effects models. This range of an individual’s true value (the value in absence of repeatability variability) would include 95% of repeated measurements for that individual at a single visit.

^d^ Repeatability is the precision of a test when replicated under identical apparent conditions (eg, same laboratory, operator, apparatus, or minimal time interval); a measure of the inherent random error associated with a test. Given an estimated 6-lead QTcF interval of 499 milliseconds (just below test cut point for clinical action) and a normal expected range of within-individual variability of ±50.2 milliseconds (ie, 2 repeatability SDs), 95% of individuals demonstrate variability between 449 and 549 milliseconds. Thus, the low positive zone for the 6-lead device is defined as the interval (500-549 milliseconds) within which a positive result could be expected to revert to negative on retest based solely on the inherent variability of the test.

^e^ The ICC is defined as the ratio of the within-individual variability to the total variability; here, this would be interpreted as the proportion of total variance that is between (rather than within) individuals. The ICC (SE) was similar whether triplicate 12-lead ECG measurements were read by 1 (0.8 [0.018]), 2 (0.822 [0.016]), or 3 (0.74 [0.043]) technical reviewers.

### Agreement of 6-Lead ECG Measurement With Reference Standard 12-Lead ECG Measurement

Assessment of intermodality agreement demonstrated a mean (SD) difference (ie, bias) between the 2 modalities of 3.7 (23.6) milliseconds (ie, the 6-lead device, on average, measured 3.7 milliseconds longer than the reference standard), with 95% limits of agreement of −50 to 43 milliseconds; 80.8% of the measurements (395 of 489) fell within a ±23.6-millisecond range (eFigure 2 in Supplement 1). Cluster analysis indicated 2 clusters of QTcF agreement data demonstrating that the 6-lead device is more likely to have a positive bias when the underlying true QTcF interval is higher (eFigure 3 in Supplement 1).

### Six-Lead ECG Device Feasibility

We surveyed BEAT Tuberculosis trial nurses (4 of 4; median years of experience, 10.5 [IQR, 7-14 years]) on the in-clinic use of the 6-lead ECG device vs conventional 12-lead ECG device (eTable 6 in Supplement 1). All nurses noted that obtaining ECG measurements, regardless of modality, was among the most time-consuming procedures for patients with RR-TB. Most nurses (3 of 4 [75.0%]) reported that the 6-lead ECG device was easy to use and that patients were more satisfied with the testing relative to the 12-lead ECG device, all nurses were confident in the results provided and felt that the 6-lead ECG device would improve clinic workflow, and most nurses (3 of 4 [75.0%]) recommended the 6-lead vs the conventional 12-lead device.

## Discussion

Longitudinal analysis of more than 2000 ECG measurements recorded on both a handheld 6-lead ECG device and a reference standard 12-lead ECG device in a resource-constrained, programmatic setting demonstrated that the 6-lead device has high NPV at clinically relevant cut points (and therefore has value as a triage test), limited PPV (related to the inherently low prevalence of prolonged QTc), and high intrinsic QTc interval variability (in practice, triplicate measurements are recommended). In addition, in this trial with concurrent administration of up to 4 QT-prolonging anti-TB drugs, 98% of patients’ QTc measurements remained below 500 milliseconds over a 6-month treatment period.

Paroxysmal ventricular fibrillation as a serious adverse drug reaction was first associated with quinidine in the 1920s,^32^ and drug-induced QT interval prolongation continues to be a major, if not enigmatic, regulatory concern.^7^ Prolongation of the QTc interval, a marker rather than a mechanism of proarrhythmia,^7,33^ is associated with increasing risk of torsade de pointes above 500 milliseconds.^6,7,8^ Among anti-TB medicines, clofazimine,^34,35,36^ the M2 metabolite of bedaquiline,^37,38,39,40^ the fluoroquinolones^41,42^ (moxifloxacin^43^ has a greater association than levofloxacin), the DM-6705 metabolite of delamanid,^26,44^ and pretomanid^19,45^ are known to prolong the QT interval,^10^ although the latter, nitroimidazoles, are considered minor QTc-prolonging agents. Despite considerable early concern around the coadministration of bedaquiline and delamanid,^46^ none of the 24 participants in the DELIBERATE trial had a QTc interval exceeding 500 milliseconds,^26^ implying no greater than additive (rather than synergistic) associations with hERG (human Ether-à-go-go-Related Gene) potassium channel blockade. In EndTB, a large phase 3 RR-TB treatment trial, QTcF intervals exceeding 500 milliseconds were infrequent and “occurred only in groups containing clofazimine and a second significant QT prolonger, bedaquiline or moxifloxacin.”^47^ In TB-PRACTECAL,^4^ another recent phase 2/3 trial, only a single patient in the experimental treatment groups (N = 214 total) discontinued treatment due to QTcF intervals exceeding 500 milliseconds. In a prospective cohort including 2296 patients treated in 16 countries,^48^ QT interval prolongation was among the least frequent clinically relevant adverse events, and in a survey of European clinicians^49^ caring for more than 1000 patients, less than 5% of patients were reported to have exceeded a QTcF interval of 500 milliseconds during treatment. In another retrospective programmatic cohort^40^ including treatment with bedaquiline, clofazimine, and levofloxacin, patients with a QTcF interval of 500 milliseconds or more (18 of 420 [4.3%]) often did not have their putative QT interval–prolonging medicines withheld, yet their QTcF interval spontaneously resolved to less than 500 milliseconds with no reported arrhythmias or related deaths.

Given such apparent reassurance in the literature in the broader context of underfunded national TB programs, the dangers of interrupting treatment of an infectious respiratory disease with high mortality, and the poorly understood predictive value of the QTc surrogate marker, it is not unreasonable to ask whether universal ECG screening and monitoring should be abandoned in place of screening only patients with specific and significant risk factors. Such has been the case with methadone maintenance programs,^50^ although not without controversy,^51^ under analogous circumstances of a potentially lethal disease with few viable treatment alternatives, busy clinics, and low risk of torsade de pointes at typical doses of methadone. However, contemporary RR-TB treatment regimens include up to 4 concurrent anti-TB QT-prolonging medicines administered to patients with common comorbidities and/or concomitant medications that themselves increase risk of torsade de pointes due to hypokalemia, impaired kidney function, age, hypothyroidism, and HIV, among other factors.^10^ Episodes of torsade de pointes have been attributed to clofazimine, bedaquiline, fluoroquinolones, and delamanid in pharmacovigilance databases (although the context of such reports is typically unknown),^36,52,53^ and there is increasing capacity to analyze these systems for causality.^54^ Several studies,^4,48,55^ including the present trial, include patients who died suddenly of a suspected cardiac cause or of an unknown cause with preceding QTc measurements exceeding 500 milliseconds. Although programmatic identification of a prolonged QTc interval has never been shown to reduce mortality in a general population, developing prospective randomized evidence supporting the superiority of specific ECG monitoring strategies or QTc interval action thresholds is implausible given the rarity of torsade de pointes and the difficulty of establishing causality.^7^ Nevertheless, several investigators^10,26,56^ have reasonably suggested ECG monitoring schedules either more targeted or less intensive than current regulatory guidelines. Simple, handheld 6-lead devices could reduce the need to perform 12-lead ECG monitoring in resource-limited settings and extend the reach of QTc assessment when needed, resulting in more patient-centered care. Such devices could also be used within planned and ongoing phase 2 and 3 TB treatment trials where mean change in QTc interval is a secondary end point.^57,58,59^

We found that the 6-lead device was easier for patients and medical staff to use than formal 12-lead ECG devices and performed well in estimating mean QTc, as in a previous assessment from a high-resource setting.^60^ At a 500-millisecond QTc interval cut point, false negatives are more problematic than false positives because the latter would theoretically trigger a formal 12-lead assessment prior to clinical management change. A widely recognized limitation of predictive values is their dependence on disease prevalence,^61^ and the inherently low prevalence of prolonged QTc intervals in the general population (and even among patients attending a heart rhythm clinic)^62^ may result in imprecise estimates of PPV. Although we did not formally assess cost in this study, a standard 12-lead ECG device (eg, from $3000 for a GE MAC 5 to $7500 for a Welch Allyn ELI 150c) costs 20 to 50 times that of a 6-lead device ($129 currently); providing automated QT interval estimations would currently incur additional costs depending on region and regulatory approvals (David Albert, MD, AliveCor, email, April 2, 2024).

### Strengths and Limitations

To our knowledge, this is the first large-scale validation of a simple, handheld 6-lead ECG device in a resource-limited setting. The strengths of our study include a strong reference standard with human-calibrated measurements and a highly representative sample closely approximating the target population. This external validity is evidenced by a low proportion (3 of 432 [0.7%]) of trial exclusions for ECG measurement abnormality or a QTcF interval greater than 450 milliseconds at baseline.^26^

This study also has some limitations, including that recordings made by each device were sequential rather than simultaneous, with the 12-lead ECG measurements recorded in the supine position and the 6-lead ECG measurements in the seated position. Consequently, heart rate variability due to positional changes may have contributed to differences between the 2 measures.^16,60,62,63^ Nevertheless, the patient positions assessed for each device reflect those recommended by the manufacturer and are likely to be encountered in clinical practice. Second, as common in RR-TB treatment trials, patients were taking QT-prolonging agents for some period prior to their baseline ECG assessment, obfuscating their true total QTcF interval increase strictly due to the trial regimens; however, the absolute QT interval duration is more informative as a marker of substantial repolarization abnormality.^64^ Third, the QTcF interval tends to undercorrect the trend of QT interval increasing as heart rate decreases with TB treatment and resolution^19,65^; results obtained through other correction methods were not statistically significantly different (eTable 7 in Supplement 1).

## Conclusion

We found that a 6-lead ECG device, with circuitry (amplifiers and filtering) and algorithm simulating that of a formal 12-lead ECG device but using an easily portable handheld platform, accurately estimated the longitudinal mean QTc interval and had high NPV for a QTc interval of 500 milliseconds or more within resource-constrained clinics. Clinical trialists and national TB programs should consider the use of handheld, 6-lead ECG devices for triage purposes and to extend the reach of 12-lead ECG monitoring when needed.

## Notes

Supplement 1.
eMethods.
**eFigure 1.** The 6-Lead ECG in Use **eFigure 2.** Bland-Altman Plot of QTcF Agreement Between the Handheld 6-Lead ECG Device and Reference Standard 12-Lead ECG **eFigure 3.** *k*-Medoids Cluster Analysis of the Differences Between the 12-Lead and Handheld 6-Lead ECG Measurements at Each Site Visit **eTable 1.** Reasons for Indeterminate QTcF Measurements **eTable 2.** Number of Conventional 12-Lead ECG and 6-Lead ECG Measurements Taken at a Single Site Visit **eTable 3.** Contingency Tables of 6-Lead QTcF vs Reference Standard 12-Lead at 500ms and 480ms Diagnostic Cutpoints **eTable 4.** Contingency Tables of 6-Lead QTcF vs Reference Standard 12-Lead at 500ms Cutpoint by Sex, Age, and BMI **eTable 5A.** Repeatability of the 12-Lead ECG QTcF Interpretation at a 500 Millisecond Cutpoint **eTable 5B.** Repeatability of the 6-Lead ECG QTcF Interpretation at a 500 Millisecond Cutpoint **eTable 6.** 12-Lead ECG and 6-Lead Handheld ECG Feasibility Survey **eTable 7.** Handheld 6-Lead and Reference Standard 12-Lead ECG QTcN and QTcB Device-Specific Parameters
eReferences.

Supplement 2.
Data Sharing Statement
