# Supplementary material for: Validation of a Handheld 6-Lead Device for QT Interval Monitoring in Resource-Limited Settings
Source: JAMA Netw Open. 2024 Jun 7;7(6):e2415576. doi: 10.1001/jamanetworkopen.2024.15576 (PMC11161846; doi:10.1001/jamanetworkopen.2024.15576)
Supplement: Supplement 1. — eMethods. eFigure 1. The 6-Lead ECG in Use eFigure 2. Bland-Altman Plot of QTcF Agreement Between the Handheld 6-Lead ECG Device and Reference Standard 12-Lead ECG eFigure 3. k-Medoids Cluster Analysis of the Differences Between the 12-Lead and Handheld 6-Lead ECG Measurements at Each Site Visit eTable 1. Reasons for Indeterminate QTcF Measurements eTable 2. Number of Conventional 12-Lead ECG and 6-Lead ECG Measurements Taken at a Single Site Visit eTable 3. Contingency Tables of 6-Lead QTcF vs Reference Standard 12-Lead at 500ms and 480ms Diagnostic Cutpoints eTable 4. Contingency Tables of 6-Lead QTcF vs Reference Standard 12-Lead at 500ms Cutpoint by Sex, Age, and BMI eTable 5A. Repeatability of the 12-Lead ECG QTcF Interpretation at a 500 Millisecond Cutpoint eTable 5B. Repeatability of the 6-Lead ECG QTcF Interpretation at a 500 Millisecond Cutpoint eTable 6. 12-Lead ECG and 6-Lead Handheld ECG Feasibility Survey eTable 7. Handheld 6-Lead and Reference Standard 12-Lead ECG QTcN and QTcB Device-Specific Parameters eReferences. [file jamanetwopen-e2415576-s001.pdf]

# Supplementary Online Content

Metcalfe JZ, Economou T, Naufal F, et al. Validation of a handheld 6-lead device for QT interval monitoring in resource-limited settings. *JAMA Netw Open.* 2024;7(6):e2415576. doi:10.1001/jamanetworkopen.2024.15576

**eMethods.**
**eFigure 1.** The 6-Lead ECG in Use
**eFigure 2.** Bland-Altman Plot of QTcF Agreement Between the Handheld 6-Lead ECG Device and Reference Standard 12-Lead ECG
**eFigure 3.** *k*-Medoids Cluster Analysis of the Differences Between the 12-Lead and Handheld 6-Lead ECG Measurements at Each Site Visit
**eTable 1.** Reasons for Indeterminate QTcF Measurements
**eTable 2.** Number of Conventional 12-Lead ECG and 6-Lead ECG Measurements Taken at a Single Site Visit
**eTable 3.** Contingency Tables of 6-Lead QTcF vs Reference Standard 12-Lead at 500ms and 480ms Diagnostic Cutpoints
**eTable 4.** Contingency Tables of 6-Lead QTcF vs Reference Standard 12-Lead at 500ms Cutpoint by Sex, Age, and BMI
**eTable 5A.** Repeatability of the 12-Lead ECG QTcF Interpretation at a 500 Millisecond Cutpoint
**eTable 5B.** Repeatability of the 6-Lead ECG QTcF Interpretation at a 500 Millisecond Cutpoint
**eTable 6.** 12-Lead ECG and 6-Lead Handheld ECG Feasibility Survey
**eTable 7.** Handheld 6-Lead and Reference Standard 12-Lead ECG QTcN and QTcB Device-Specific Parameters
**eReferences.**

This supplementary material has been provided by the authors to give readers additional information about their work.

© 2024 Metcalfe JZ et al. *JAMA Network Open.*

**eMethods.**

<u>Statistical analyses</u>

Heart rate, QT interval, and QTc were abstracted from reference standard 12-lead (i.e., manually read Clario measurements, as above) and automated/algorithmic handheld 6-lead ECG measurements. QTc was calculated using the Fridericia formula ($QTc = QT / RR^{1/3}$);[1] we further assessed a population-specific (QTcN)[2] and Bazett's ($QTcB = QT / RR^{1/2}$) correction method (eTable 1). Mean increase in QTc was measured using a linear mixed effects regression model of QTc on clinic visit with random intercepts by participant. Clinic visits with fewer than triplicate 12-lead reference standard QTcF measurements were excluded from the primary analysis. Visit-level triplicate (or more, if more than three measurements were available) QTcF measurements for both devices were averaged, compared, and illustrated using scatter plots.[3] Outlier 12-lead reference standard QTcF measurements (>40ms range between triplicate measurements) were rechecked and re-analyzed if appropriate by an adjudicating cardiologist (co-author, RK). In accordance with prior studies,[4,5] we reported the maximum mean change in QTc from baseline.

Quantitative inter-modality agreement was measured using Bland-Altman plots.[6] The *k*-medoids clustering technique was used to group the results of the Bland Altman plot into two clusters. We utilized this method instead of the *k*-means to reduce distortion from outliers. We used the Within-Sum-of-Squares (WSS) or Elbow method[7,8] to identify the ideal number of clusters; this method is used in cluster analysis to determine the optimal number of clusters by identifying the point at which the addition of another cluster does not significantly improve the fit of the model, visually resembling an "elbow" on the plot. A contingency table was used to describe the performance of the QTcF classification model using the FDA-suggested cutpoints of 500ms and 480ms.[9–11] We assessed the repeatability of each modality using a linear mixed-effects model fit to the numerical QTcF values. Such models allow global estimation of within-person standard deviation (SD) optimally weighted for the correlation structure of the repeated measurements.[12,13] Assuming that 95% of

© 2024 Metcalfe JZ et al. *JAMA Network Open.*

measurements are located within 1.96 SDs, the “normal expected range” of within-subject test repeatability can be calculated by expressing this SD as a percentage of the individual’s mean QTcF. Thus, given an underlying ‘true’ mean test result, linear mixed effect models estimate the expected variability that will occur with repeat measurement; the resultant standard deviation is thus an estimate of how close a given measurement is to the true value. As test-retest differences were not normally distributed, the 12-lead and 6-lead datasets were verified of having 95% of differences contained within 1.96 SDs of their means by a resampling procedure using 10,000 bootstrap iterations. We conducted a qualitative survey of nurses who performed the 12-lead and handheld 6-lead measurements assessing ease of use and preferences for use in clinical workflow. All P values were two-sided with alpha=0.05 as the significance level. Data analysis was performed using Python 3.11.1 (Python, Scotts Valley, CA) and Stata 15.1 (Stata Corporation, College Station, TX).

© 2024 Metcalfe JZ et al. *JAMA Network Open.*

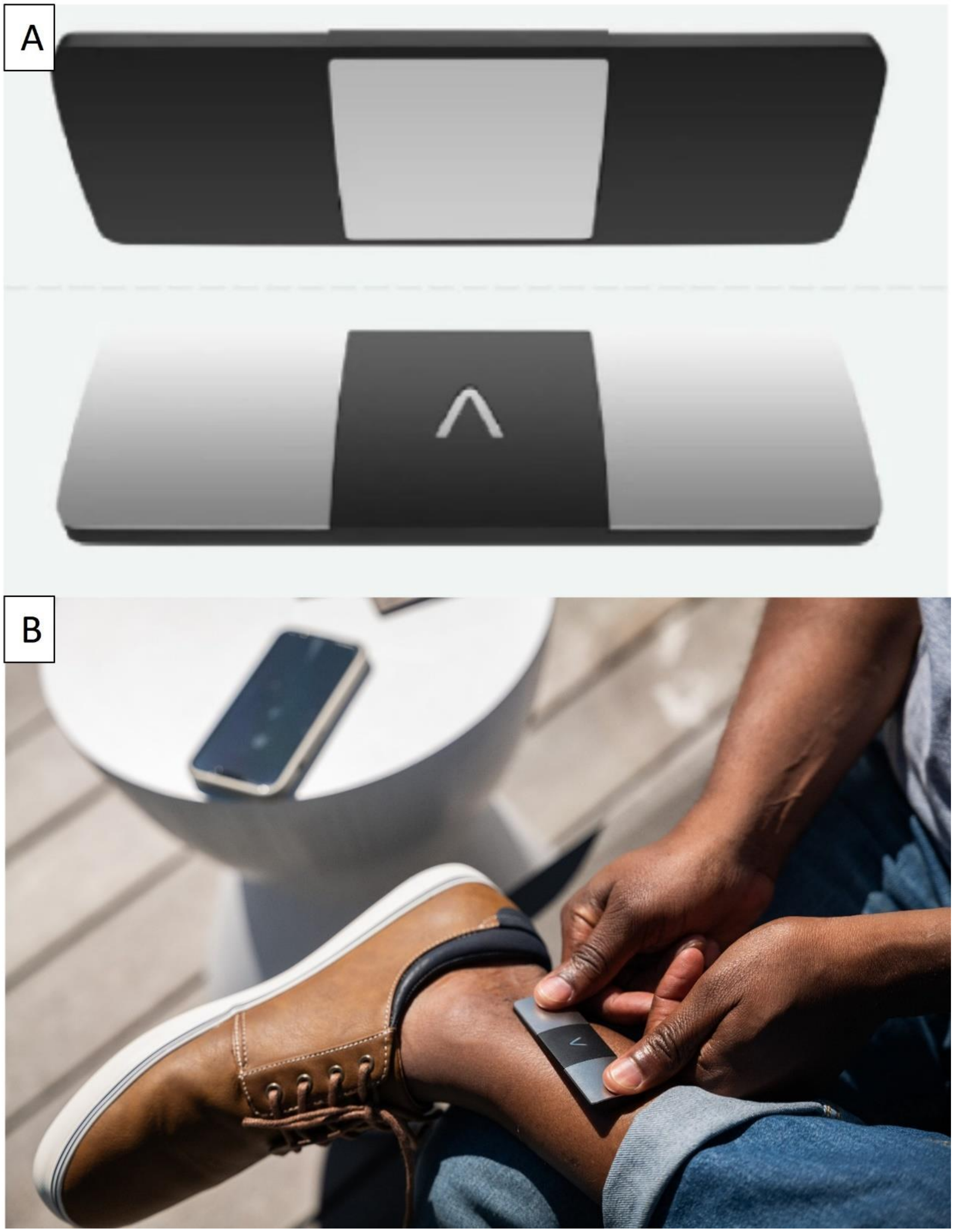


**eFigure 1.** The 6-Lead ECG in Use

A, This panel illustrates the hand-held AliveCor KardiaMobile 6L from the bottom and top.
B, This panel shows one of the recommended methods of using the six-lead device. Both thumbs are placed on the two top electrodes and the third bottom electrode is in contact with the skin above the left ankle (the bottom electrode can also be in contact with the skin above the left knee).

© 2024 Metcalfe JZ et al. *JAMA Network Open.*

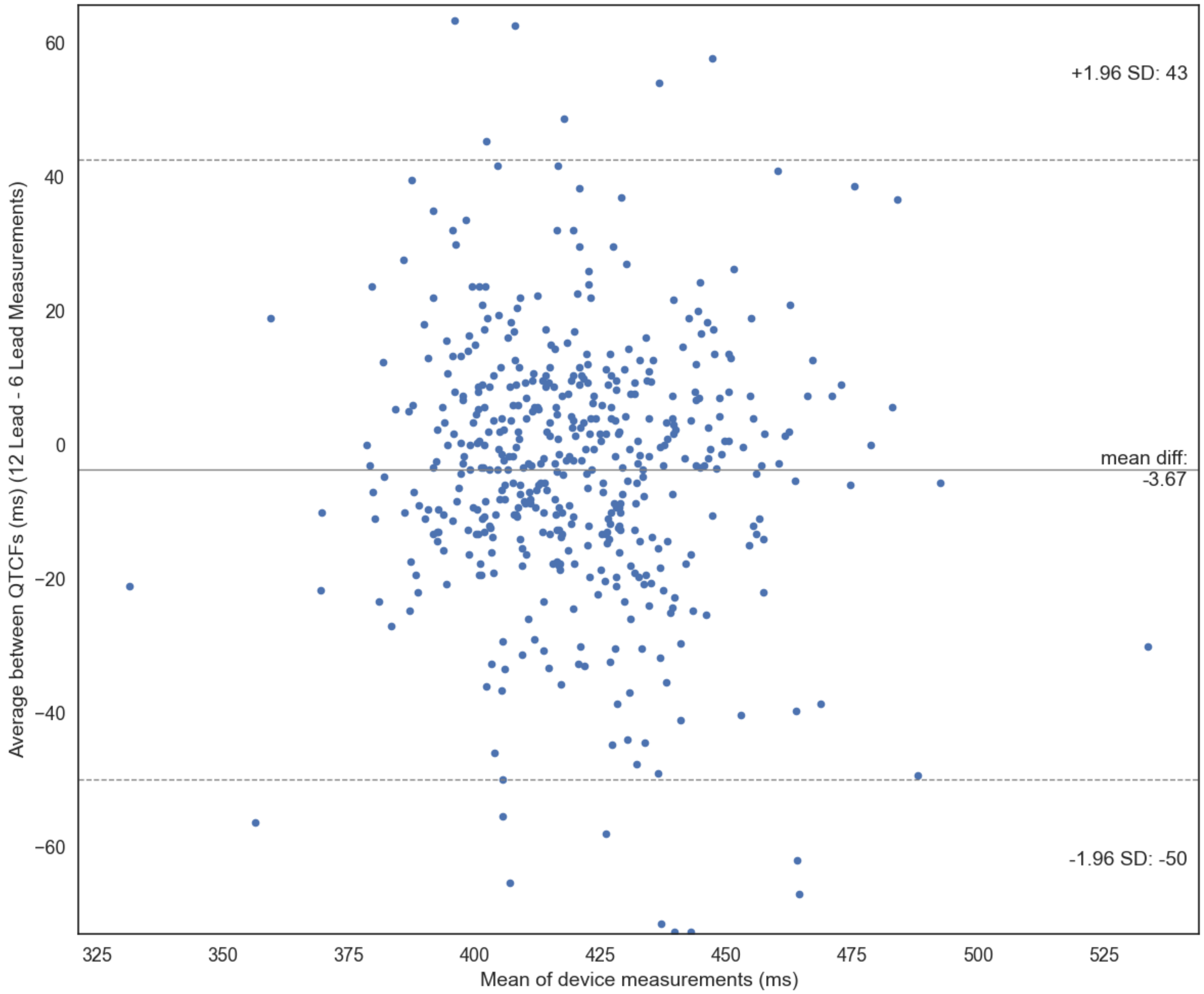


**eFigure 2.** Bland-Altman Plot of QTcF Agreement Between the Handheld 6-Lead ECG Device and Reference Standard 12-Lead ECG

Bland-Altman analysis for the triplicate averaged QTcF measurements (6-lead vs. 12-lead) across full range of QTcF values for 489 individual comparisons across 170 participants showed a mean difference of -3.7 milliseconds (ms) and 1.96 standard deviations (1.96 SD) of ± 46.3ms. The lower and upper limits of agreement (mean difference ± 1.96 SD) are +43ms and -50ms, respectively.

© 2024 Metcalfe JZ et al. *JAMA Network Open.*

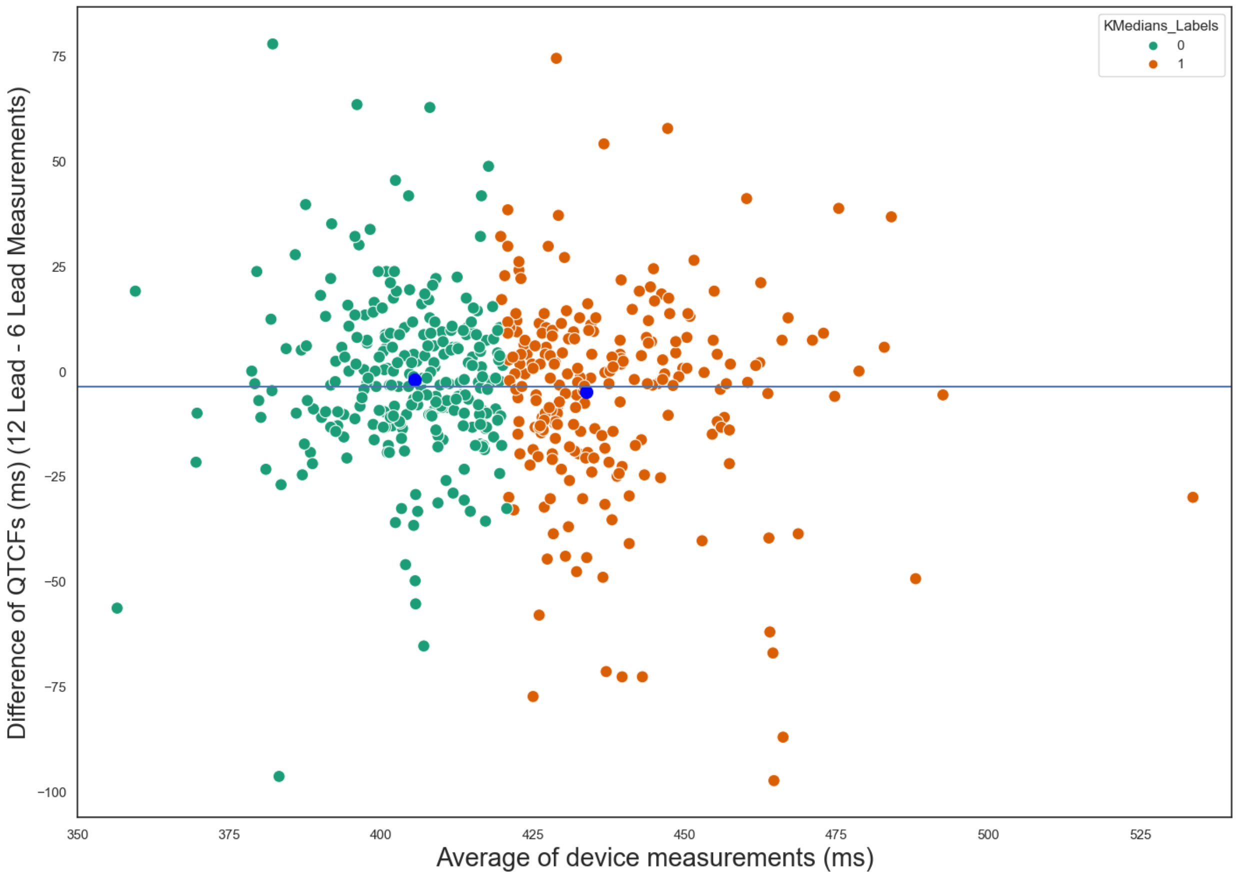


**eFigure 3.** *k*-Medoids Cluster Analysis of the Differences Between the 12-Lead and Handheld 6-Lead ECG Measurements at Each Site Visit

The *k*-medoids clustering technique was used to group the results of the Bland Altman plot into two clusters. We utilized this method instead of the *k*-means to reduce distortion from outliers. To identify that two clusters were the ideal number of clusters, we used the Within-Sum-of-Squares/Elbow method. The cluster centers are illustrated by two blue circles, and the bias (-3.7ms) from the Bland Altman analysis is shown by a horizontal blue line. The cluster centers can clearly be seen to be above and below the bias, indicating that the 6-lead tended to underestimate and overestimate QTcF when the actual QTcF is low or high respectively.

© 2024 Metcalfe JZ et al. *JAMA Network Open.*

| | Reasons for indeterminate QTcF measurement |
|---|---|
| 12-lead ECG QTcF assessments (N=2,070) | 43 indeterminate (2.1%)<br>• 33 Normal rhythm strip<br>• 5 Abnormal rhythm strip<br>• 5 Unable to Evaluate rhythm strip |
| 6-lead ECG QTcF assessments (N=2,015) | 235 indeterminate (11.7%)<br>• 75 Unreadable rhythm strip<br>• 76 Unclassified rhythm<br>• 36 Wide QRS<br>• 31 Atrial fibrillation<br>• 17 Reading too short to apply algorithm |

**eTable 1.** Reasons for Indeterminate QTcF Measurements

Over the study duration, 2,070 and 2,015 total 12-lead and 6-lead ECG assessments were made, 2.1% and 11.7% of which were indeterminate, respectively. The table reflects all indeterminate QTcF measurements, regardless of number of valid measures ultimately obtained at individual clinic visits.

© 2024 Metcalfe JZ et al. *JAMA Network Open.*

| | | Number of 12-lead ECG replicates taken at a single site visit | | | | | |
|---|---|---|---|---|---|---|---|
| | | **>3** | **Triplicate** | **Duplicate** | **Single** | **None** | **Total** |
| **Number of 6-lead ECG replicates taken at a single site visit** | **>3** | - | 21 | 1 | - | - | 22 |
| | **Triplicate** | 1 | 467 | 24 | 4 | 6 | 502 |
| | **Duplicate** | - | 68 | 4 | - | - | 72 |
| | **Single** | - | 40 | 1 | - | - | 41 |
| | **None** | - | 54 | 2 | 3 | 1 | 60 |
| | **Total** | 1 | 650 | 32 | 7 | 7 | 697 |

**eTable 2.** Number of Conventional 12-Lead ECG and 6-Lead ECG Measurements Taken at a Single Site Visit

(1) 60 site visits had no measurements taken using the 6-lead ECG handheld device, while 7 visits had no available measurements with the 12-lead ECG.

(2) 1 site visit had more than 3 measurements taken using the 12-lead ECG. 22 site visits had more than 3 measurements taken using the 6-lead ECG.

© 2024 Metcalfe JZ et al. *JAMA Network Open.*

| | | | | | | |
|---|---|---|---|---|---|---|
| **Duplicate and single 6-Lead measurements only** | 6-Lead | 12-Lead | | | | |
| | | *Spearman Rho = 0.34, r2 = 0.11, p = 0.004* | | | | |
| | | | 500 ms cut point | | 480 ms cut point | |
| | | | Abnormal | Normal | Abnormal | Normal |
| | | Abnormal | 0<br>TP | 4<br>FP | 0<br>TP | 7<br>FP |
| | | Normal | 2<br>FN | 102<br>TN | 2<br>FN | 99<br>TN |

**eTable 3.** Contingency Tables of 6-Lead QTcF vs Reference Standard 12-Lead at 500ms and 480ms Diagnostic Cutpoints
TN = True negative; FN = False negative; TP = True positive; FP = False positive.

The contingency tables display the number of true negative, false negative, true positive, and false positive measurements with the triplicate averaged 12-lead ECG QTcF measurements as the reference standard and all handheld 6-lead ECG QTcF measurements as the predictor. Relative to the primary analysis shown in Figure 1 (including only triplicate or greater 6-lead measurements), correlation and cutpoint classification were less favorable when fewer than triplicate 6-lead measurements were analyzed against reference standard triplicate 12-lead ECG QTcF.

© 2024 Metcalfe JZ et al. *JAMA Network Open.*

| Sex | 6-Lead | 12-Lead | | | | |
|---|---|---|---|---|---|---|
| | | | Males | | Females | |
| | | | Abnormal | Normal | Abnormal | Normal |
| | | Abnormal | 1<br>TP | 2<br>FP | 0<br>TP | 3<br>FP |
| | | Normal | 1<br>FN | 267<br>TN | 0<br>FN | 215<br>TN |

| Age | 6-Lead | 12-Lead | | | | |
|---|---|---|---|---|---|---|
| | | | Age <50 | | Age >=50 | |
| | | | Abnormal | Normal | Abnormal | Normal |
| | | Abnormal | 1<br>TP | 4<br>FP | 0<br>TP | 1<br>FP |
| | | Normal | 0<br>FN | 410<br>TN | 1<br>FN | 72<br>TN |

| BMI | 6-Lead | 12-Lead | | | | |
|---|---|---|---|---|---|---|
| | | | Underweight | | Normal BMI | |
| | | | Abnormal | Normal | Abnormal | Normal |
| | | Abnormal | 0<br>TP | 3<br>FP | 1<br>TP | 0<br>FP |
| | | Normal | 0<br>FN | 191<br>TN | 1<br>FN | 226<br>TN |

**eTable 4.** Contingency Tables of 6-Lead QTcF vs Reference Standard 12-Lead at 500ms Cutpoint by Sex, Age, and BMI

BMI = body mass index

The contingency tables display the number of true negative, false negative, true positive, and false positive measurements with the triplicate averaged 12-lead ECG QTcF measurements as the reference standard. Differential effects of sex, age (less than 50 compared to equal to or over 50), and BMI (underweight [<=18.5] compared to normal [>18.5 and <25]) are shown. There were no positive measurements among female participants at the 500 ms cutpoint, but PPV among males was 33.3% (95% CI, 6.6% to 78.0%). Among participants 50 years of age and older, there was only one measurement above 500ms (false positive) by the 6-lead device and none by the 12-lead. PPV in those under the age of 50 was 20% (95% CI, 8.6% to 39.9%). There were three false positives among BMI underweight participants and no true positives. Among normal weight participants, there was one true positive measurement and no positive measurements by the 6-lead. Negative predictive values were similar (98.6-100%) across all groups.

© 2024 Metcalfe JZ et al. *JAMA Network Open.*

| Test Result Sequence | Total clinic time points (N=692) |
|---|---|
| **Concordant** | **660 (95.3%)** |
| Negative/Negative | 12 |
| Negative/Negative/Negative | 640 |
| Positive/Positive/Positive | 2 |
| Indeterminate/Indeterminate/Indeterminate | 5 |
| Negative/Negative/Negative/ Negative/Negative/Negative | 1 |
| | |
| **Discordant** | **32 (4.6%)** |
| Indeterminate/Negative | 1 |
| Negative/Negative/Positive | 1 |
| Negative/Negative/Indeterminate | 5 |
| Negative/Positive/Negative | 1 |
| Negative/Positive/Positive | 2 |
| Negative/Indeterminate/Negative | 5 |
| Negative/Indeterminate/Indeterminate | 1 |
| Positive/ Negative/Negative | 1 |
| Positive/ Positive /Negative | 2 |
| Indeterminate/ Negative/Negative | 10 |
| Indeterminate/ Negative/Indeterminate | 2 |
| Negative/ Indeterminate /Negative/ Negative | 1 |

**eTable 5A.** Repeatability of the 12-Lead ECG QTcF Interpretation at a 500 Millisecond Cutpoint

'Positive' results are measurements greater than 500 ms. Indeterminate results have no QTcF measurements available. Three additional clinic time points (not shown) had only one attempt; all of these attempts had 'Negative' (i.e., QTcF <500 ms) results. One time point had no 12-lead measurements but did have 6-lead measurements and one visit had no measurements from either device. Visits with concordant measures were 98.8% (643/651) of all visits with at least three determinate 12-lead measurements.

© 2024 Metcalfe JZ et al. *JAMA Network Open.*

| Test Result Sequence | Total visits (N=659) |
|---|---|
| **Concordant** | **513 (77.8%)** |
| Negative/Negative | 4 |
| Indeterminate/Indeterminate | 2 |
| Negative/Negative/Negative | 466 |
| Positive/Positive/Positive | 3 |
| Indeterminate/Indeterminate/Indeterminate | 19 |
| Negative/Negative/Negative/Negative | 17 |
| Positive/Positive/Positive/Positive | 1 |
| Indeterminate/Indeterminate/Indeterminate/Indeterminate | 1 |
| | |
| **Discordant** | **146 (22.1%)** |
| Negative/Negative/Positive | 8 |
| Negative/Negative/Indeterminate | 18 |
| Negative/Positive/Negative | 10 |
| Negative/Positive/Positive | 1 |
| Negative/Indeterminate/Negative | 23 |
| Negative/Indeterminate/Indeterminate | 11 |
| Positive/Negative/Negative | 4 |
| Positive/Negative/Positive | 1 |
| Positive/Indeterminate/Indeterminate | 2 |
| Indeterminate/Negative/Negative | 22 |
| Indeterminate/Negative/Indeterminate | 11 |
| Indeterminate/Positive/Negative | 1 |
| Indeterminate/Positive/Indeterminate | 1 |
| Indeterminate/Indeterminate/Negative | 11 |
| Indeterminate/Indeterminate/Positive | 1 |
| Negative/Negative/Negative/Positive | 1 |
| Negative/Indeterminate/Negative/Negative | 1 |
| Negative/Indeterminate/Indeterminate/Negative | 1 |
| Negative/Indeterminate/Indeterminate/Indeterminate | 1 |
| Positive/Positive/Negative/Positive | 2 |
| Indeterminate/Negative/Negative/Negative | 7 |
| Indeterminate/Negative/ Indeterminate /Negative | 1 |
| Indeterminate/Indeterminate /Negative/Negative | 2 |
| Indeterminate/Indeterminate/Negative/Indeterminate | 1 |
| Indeterminate/Indeterminate/Indeterminate/Negative | 2 |
| Negative/Negative/Negative/ Negative/Negative/Indeterminate | 1 |
| Indeterminate / Indeterminate / Indeterminate / Negative/Negative/ Negative | 1 |

**eTable 5B.** Repeatability of the 6-Lead ECG QTcF Interpretation at a 500 Millisecond Cutpoint

Positive results are measurements above a 500 ms cutpoint. Indeterminate results have no QTcF measurements available. Thirty-seven visits had no 6-lead measurements but did have 12-lead measurements and one visit had no measurements from either device. Visits with concordant measures were 92.9% (487/524 visits) of all visits with at least three determinate 6-lead measurements.

© 2024 Metcalfe JZ et al. *JAMA Network Open.*

| Question | Response (n=4) |
| --- | --- |
| **Sex** | 100% Female |
| **Age** | |
| Median (range) — yr | 40.5 [36 – 44] |
| **Years of experience** | |
| Median (range) — yr | 10.5 [1 – 20] |
| **Did you conduct electrocardiogram tests using the KardiaMobile 6L hardware?** | |
| No | 0 (0%) |
| Yes | 4 (100%) |
| **How easy is it to use the KardiaMobile 6L ?** | |
| 1-Very difficult | 0 (0%) |
| 2-Somewhat difficult | 0 (0%) |
| 3-Neither difficult nor easy | 1 (25%) |
| 4-Somewhat easy | 2 (50%) |
| 5-Very easy | 1 (25%) |
| **How easy is it to use the KardiaMobile application on the smartphone?** | |
| 1-Very difficult | 0 (0%) |
| 2-Somewhat difficult | 0 (0%) |
| 3-Neither difficult nor easy | 0 (0%) |
| 4-Somewhat easy | 3 (75%) |
| 5-Very easy | 1 (25%) |
| **How confident were you in the results provided by the KardiaMobile 6L ?** | |
| 1-Not very confident | 0 (0%) |
| 2-Somewhat not confident | 0 (0%) |
| 3-Neither not confident nor confident | 0 (0%) |
| 4-Somewhat confident | 4 (100%) |
| 5-Very confident | 0 (0%) |
| **How easy is it to use the Conventional 12-lead ECG ?** | |
| 1-Very difficult | 0 (0%) |
| 2-Somewhat difficult | 0 (0%) |
| 3-Neither difficult nor easy | 2 (50%) |
| 4-Somewhat easy | 1 (25%) |
| 5-Very easy | 1 (25%) |
| **How confident were you in the results provided by the Conventional 12-lead ECG?** | |
| 1-Not very confident | 0 (0%) |
| 2-Somewhat not confident | 1 (25%) |
| 3-Neither not confident nor confident | 0 (0%) |
| 4-Somewhat confident | 3 (75%) |
| 5-Very confident | 0 (0%) |
| **How likely are you to recommend the use of the KardiaMobile 6L over conventional 12-lead ECG?** | |
| 1-Very unlikely | 0 (0%) |
| 2-Somewhat unlikely | 0 (0%) |
| 3-Neither unlikely nor likely | 1 (25%) |
| 4-Somewhat likely | 2 (50%) |
| 5-Very likely | 1 (25%) |
| **How was the satisfaction of your patients when undergoing testing with the KardiaMobile 6L system, compared to the conventional 12-lead ECG?** | |
| 1-Very dissatisfied | 0 (0%) |
| 2-Somewhat dissatisfied | 0 (0%) |
| 3-Neither dissatisfied nor satisfied | 1 (25%) |
| 4-Somewhat satisfied | 2 (50%) |
| 5-Very satisfied | 1 (25%) |
| **In general, how time consuming of a procedure is measuring ECGs for drug-resistant TB patients, compared to other procedures that must be done at each visit?** | |
| 1-Hard, one of the most time-consuming parts of the visit | 1 (25%) |
| 2-Time-consuming, but not the most time-consuming | 3 (75%) |
| 3-About average difficulty for all procedures that need to be performed | 0 (0%) |
| 4-Not very time-consuming | 0 (0%) |
| 5-Easy, one of the least time-consuming parts of the visit | 0 (0%) |
| **How would using KardiaMobile 6L instead of the standard 12-lead ECG impact your clinic workflow?** | |

© 2024 Metcalfe JZ et al. *JAMA Network Open.*

| | |
|---|---|
| Response 1 | "12 lead is time consuming since participant has to undress so 6L would assist in patient flow." |
| Response 2 | "Will definitely improve the clinic workflow." |
| Response 3 | "Reduce patient clinic time." |
| Response 4 | "It would assist with time constraints making workflow much faster, also patient not having to undress especially when it's very cold weather." |
| **Did you have any reasons for not taking or being able to take a measurement with the KardiaMobile 6L following the conventional 12-lead ECG ? Please explain.** | |
| Response 1 | *No response* |
| Response 2 | "Depended on the participant's condition and if participant is shaking too much, though done on scheduled visit" |
| Response 3 | "No" |
| Response 4 | "No" |

**eTable 6.** 12-Lead ECG and 6-Lead Handheld ECG Feasibility Survey

All (4/4; 2 nurses per site) BEAT-TB tuberculosis nurses queried responded to the survey. Obtaining ECG measurements for RR-TB patients were noted to be time-consuming by all nurses. None reported difficulty with using the 6-lead device or the smartphone-based application. Most (75%) were more satisfied with the 6-lead ECG testing relative to the 12-lead ECG, and all were confident in the results provided and felt that the 6-lead device would improve clinic workflow. Most (75%) recommended the 6-lead over conventional 12-lead device. One nurse noted that a patient's condition may impact ability to measure using the 6-lead device.

© 2024 Metcalfe JZ et al. *JAMA Network Open.*

| | 12-lead ECG | 6-lead ECG |
|---|---|---|
| **Individual device parameters - QTcN** | | |
| *N* | 651 | 524 |
| Normal Expected Range[a] | +/- 24.0 | +/- 49.8 |
| Repeatability SD (ms)[b] | +/- 12.2 | +/- 25.4 |
| ICC[c] (SE) | 0.8<br>(0.02) | 0.5<br>(0.03) |
| **Individual device parameters - QTcB** | | |
| *N* | 651 | 524 |
| Normal Expected Range[a] | +/- 25.4 | +/- 49.8 |
| Repeatability SD (ms)[b] | +/- 13.0 | +/- 25.4 |
| ICC[c] (SE) | 0.8<br>(0.02) | 0.5<br>(0.03) |

**eTable 7.** Handheld 6-Lead and Reference Standard 12-Lead ECG QTcN and QTcB Device-Specific Parameters

SD = standard deviation; ms = milliseconds; ICC = Intraclass correlation coefficient.
Individual device parameters were calculated for each device from visits including triplicate averaged measurements, regardless of measurement availability for the other device. All statistics except for *N* are presented in milliseconds.

[a] Normal expected range of within-subject variability, estimated using linear mixed effects models. This range about an individual's true value (the value in absence of repeatability variability) would include 95% of repeat measurements for that individual at a single visit.

[b] Repeatability is the precision of a test when replicated under identical apparent conditions (e.g., same laboratory, operator, apparatus, minimal time interval); a measure of the inherent random error associated with a test.

Given an estimated 6-lead QTcN or QTcB of 499 ms (just below test cutpoint for clinical action) and a normal expected range of within-subject variability of +/- 49.8 ms (i.e., two repeatability standard deviations), 95% of subjects demonstrate variability between 449 and 549 ms. Thus, the low positive zone for the 6-lead device is defined as the interval (500-549 ms) within which a positive result could be expected to revert to negative on retest based solely on the inherent variability of the test.

[c] The intraclass correlation coefficient is defined as the ratio of the within-person variability to the total variability; here, this would be interpreted as the proportion of total variance that is between (rather than within) individuals.

© 2024 Metcalfe JZ et al. *JAMA Network Open.*

**eReferences.**


1. Fridericia LS. The duration of systole in an electrocardiogram in normal humans and in patients with heart disease. *Ann Noninvasive Electrocardiol*. 2003;8(4):343-351. doi:10.1046/J.1542-474X.2003.08413.X
2. Li H, Salinger DH, Everitt D, et al. Long-term effects on QT prolongation of pretomanid alone and in combinations in patients with tuberculosis. *Antimicrob Agents Chemother*. 2019;63(10). doi:10.1128/AAC.00445-19/SUPPL_FILE/AAC.00445-19-S0001.PDF
3. Everitt BS, Fleiss JL. *Statistical Methods for Rates and Proportions.* 2nd ed. Wiley; 1981. doi:10.2307/2530193
4. Wallenstein G, Walter B, Fritsch H, Taube T. Strategic and Statistical Considerations on the QT Assessment of Volasertib. *https://doi.org/101177/2168479017739826*. 2017;52(4):416-422. doi:10.1177/2168479017739826
5. Kleiman RB, Darpo B, Thorn M, Stoehr T, Schippers F. Potential strategy for assessing QT/QTc interval for drugs that produce rapid changes in heart rate: Electrocardiographic assessment of the effects of intravenous remimazolam on cardiac repolarization. *Br J Clin Pharmacol*. 2020;86(8):1600. doi:10.1111/BCP.14270
6. Martin Bland J, Altman DG. Statistical Methods for Assessing Agreement between two Methods of Clinical Measurement. *The Lancet*. 1986;327(8476):307-310. doi:10.1016/S0140-6736(86)90837-8
7. Kaufman L, Rousseeuw PJ. *Partitioning Around Medoids (Program PAM)*. John Wiley & Sons, Ltd; 1990. doi:10.1002/9780470316801.CH2
8. Ketchen D, journal CS. The application of cluster analysis in strategic management research: an analysis and critique. *Strategic Management Journal*. 1996;17(6):441-458.
9. FDA. Guidance for Industry - E14 Clinical Evaluation of QT/QTc Interval Prolongation and Proarrhythmic Potential for Non-Antiarrhythmic Drugs. Published online 2005.
10. Zeppenfeld K, Tfelt-Hansen J, De Riva M, et al. 2022 ESC Guidelines for the management of patients with ventricular arrhythmias and the prevention of sudden cardiac death. *Eur Heart J*. 2022;43(40):3997-4126. doi:10.1093/EURHEARTJ/EHAC262
11. Dooley KE, Rosenkranz SL, Conradie F, et al. QT Effects Of Bedaquiline, Delamanid Or Both In Patients With Rifampicin-Resistant-TB: A Randomized Controlled Trial. *Lancet Infect Dis*. 2021;21(7):975. doi:10.1016/S1473-3099(20)30770-2
12. Laird NM, Ware JH, Lairdl NM, Warel JH. Random-effects models for longitudinal data. *Biometrics*. 1982;38(4):963-974.
13. Diggle PJ. An approach to the analysis of repeated measurements. *Biometrics*. Published online 1988:959-971.


© 2024 Metcalfe JZ et al. *JAMA Network Open.*
