# Supplementary material for: Validation of a Handheld 6-Lead Device for QT Interval Monitoring in Resource-Limited Settings
Source: JAMA Netw Open. 2024 Jun 7;7(6):e2415576. doi: 10.1001/jamanetworkopen.2024.15576 (PMC11161846; doi:10.1001/jamanetworkopen.2024.15576)
Supplement: Supplement 2. — Data Sharing Statement [file jamanetwopen-e2415576-s002.pdf]

Data Sharing Statement

Metcalfe. Validation of a Handheld 6-Lead Device for QT Interval Monitoring in Resource-Limited Settings. *JAMA Netw Open*. Published June 07, 2024.
doi:10.1001/jamanetworkopen.2024.15576

Data
Data available: Yes
Data types: Deidentified participant data
How to access data: john.metcalfe@ucsf.edu
When available: With publication

Supporting Documents
Document types: None

Additional Information
Who can access the data: Researchers whose proposed use of the data has been approved
Types of analyses: For any purpose
Mechanisms of data availability: Signed data access agreement
